# Retrospective cross-sectional study of asthma severity in adult patients at the Jimma Medical Center, Ethiopia

**DOI:** 10.1038/s41598-022-15807-1

**Published:** 2022-07-07

**Authors:** Desalew Tilahun, Mesay Michael, Mihret Gashaye, Eneyew Melkamu, Tsiyon Mekoya

**Affiliations:** 1 School of Nursing, Institute of Health, Jimma University, Jimma, Ethiopia; 2 Institute of Health, Jimma University, Jimma, Ethiopia; 3 School of Midwifery, Institute of Health, Jimma University, Jimma, Ethiopia

**Keywords:** Diseases, Health care

## Abstract

Asthma is one of the most prevalent chronic diseases and is a public health problem worldwide. It is a long-standing condition affecting the respiratory system. Thus this study aimed to assess the severity of asthma in patients at the adult emergency department of Jimma Medical Center (JMC), Southwest Ethiopia. A one year (1 May, 2020, to 1 May, 2021) retrospective cross-sectional study was conducted among 189 patients at the adult emergency department of JMC. Data were collected between 25 July, 2021 to 25 August, 2021 by two Bachelor of Science degree holders in nursing (BSC) nurses after providing proper training. We used structured checklist that was obtained from previous studies to collect the data. Finally, data were entered into EpiData version 3.1 then exported to Stata version 15.0 for further analysis. Multinomial analysis was used to estimate odds ratios (OR) and 95% confidence intervals (CI) for the association between risk factors and severity of asthma. Of 195 patients retrieved from the Health management information system (HMIS) logbook and patient profile, 189 fulfilled the eligibility criteria giving a response rate of 96.9%. The mean age of patients was 47.69 (± 19.02) years old ranging from 20 to 85. More than one third of the patients were age range of 20–39 years. Only more than half of the patients were women. Almost 46% of the patients had moderate asthma. Being male, merchant and government employees had lower odds of asthma than their counterparts whereas being daily laborers and smoking contributed to increased odds of moderate asthma. Patients’ age and comorbidities had increased odds of severe asthma in relation to the participants of their reference category. Urban residents had decreased odds of severe asthma compared to their rural counterparts. This study highlights that majority of patients had moderate asthma. Health care providers should pay special attention to accurately diagnosing asthma according to its severity which is essential to the optimal management of asthma. This study calls JMC health care providers to give due attention while providing routine care for their patients in accordance to identified factors.

## Introduction

Asthma is the most common chronic respiratory disease worldwide^1,2^. Chronic respiratory diseases have become a public health challenge in high-income countries owing to their recurrence rates and financial effects^3^. Globally, asthma is one of the main causes of chronic morbidity and mortality which is leading to a huge economic and social burden^3^ and affecting people of all ages, sexes, and ethnicities^4^. The global prevalence of asthma ranges from 1 to 18% in different studies^5^. In Africa, asthma is a neglected disease and its prevalence is estimated at an average of 12% with national estimates ranging from 2 to 53% among individuals aged < 2 to 64 years^5^. The prevalence of asthma varies across Africa, with 6.9% in Kinshasa^6^, 1.5% in Nigeria^7^, 29.6% in Debre Berhan Referral Hospital in Ethiopia^8^, 1.5% in Zewditu Memorial hospital in Ethiopia^9^.

The important components used to manage asthmatic patients are assessment, monitoring, health education, regulating environmental factors and co-morbid situations that contribute to allergies and severity^10,11^.The worldwide prevalence of asthma across the globe is influenced by age,gender,residence,occupational status, comorbidity, smoking history, and family history of diseases^8,12–16^.

Asthma continues to be a major source of the global economic burden in terms of both direct and indirect costs. Given that asthma cannot be cured but can be controlled, attempts to reduce costs should focus on better disease management. This approach is consistently associated with a significant reduction in asthma costs. Accurate diagnosis in terms of severity, improved access to care, especially to controller (including preventer) therapies, and better adherence to such therapies can significantly reduce the economic burden of asthma resulting in good asthma control and quality of life^17^.Thus, health education is considered an important factor in meeting the challenges of asthma in Africa^18^. Several studies have extensively demonstrated that the prevalence of asthma is variable. However, little has been investigated about the severity of asthma, as to our knowledge, which is paramount in diagnosing and managing the disease appropriately. Thus, the current study aimed to determine the severity of asthma in adult patients at the Adult Emergency Department of the JMC, Ethiopia, in a retrospective cross-sectional study.

## Materials and methods

### Study design and setting

This hospital-based retrospective cross-sectional study was conducted in the adult emergency department of, JMC from 25 July, 2021 to 25 August, 2021. JMC is found in Jimma town which is 354 km from the capital Addis Ababa within the southwest of Ethiopia. JMC is one of the oldest public hospitals that provides inpatient, outpatient, and emergency and chronic clinic follow-up services for an estimated 15 million people in the southwest part of the country. The total number of patients who visited the adult emergency unit of JMC from 1 May, 2020, to 1 May, 2021 was 11,235. Among these, 195 were diagnosed with asthma.

### Study participants and sample size

All adults aged 18 years or older with asthma admitted to the emergency department of JMC from 1 May, 2020, to 1 May, 2021, with clear documented information on the diagnosis of asthma during the study period were included. There was no sample size calculation for this study as the number of participants was small which 195 we used the census.

A census is a collection of information from all units in the population or a 'complete enumeration' of the population. We use a census method when we want accurate information for many subdivisions of the population, such as data for small areas may be available and data for sub-populations may be available, assuming satisfactory response rates are achieved because of these reasons detailed cross-tabulations may be possible.

### Data collection instrument and procedure

The questionnaire was designed in two parts which included socio-demographic data (age, gender, residence, occupational status, comorbidity (pneumonia, allergic rhinitis, COPD and CVD), and smoking status) and clinical presentation of asthma. First, patients with a diagnosis of asthma were retrieved from the HMIS log book, and the data were collected by gathering all medical records of patients with asthma using the census method for all patients aged ≥ 18 years. Data were collected by the researcher and two properly trained BSC nurses. The required data were obtained following a structured checklist used in previous works of the group.

### Operational definition

#### Mild asthma

If the speech was in sentences, accessory muscle was not used, respiratory rate was 16 to 20 breaths per minute, heart rate was 80 to 100 beats per minute, oxygen saturation was ≥ 95%.

#### Moderate asthma

If the speech was in phrases, mental status was agitated, sometimes use of accessory muscles, respiratory rate was 20 to 30 breaths per minute, heart rate was 100 to 120 beats per minute, and oxygen saturation was 90–95%.

#### Severe asthma

If the speech was in words, mental status was distressed, used accessory muscles, respiratory rate > 30 breaths per minute, heart rate > 120 beats per minute, and oxygen saturation < 90%^9^.

### Data quality control

Data quality was ensured before, during and after the data collection. Before data collection, the validity of the questionnaire was checked by experts. Training was provided to the data collectors for common understanding by the investigator. During the data collection period, the purpose of data collection and the importance of the study were informed to the card keepers to generate quality data.

There was close supervision during the data collection.The collected data were checked for completeness and consistency by the investigator daily basis and necessary corrective actions were made accordingly.

### Data processing and analysis

The collected data were entered into EpiData version 3.1 then exported into Stata version 15.0 for further analysis. Descriptive analyses (frequencies, percentages, means and standard deviations) were computed to explore socio-demographic, clinical presentations of asthma and the severity of asthma, and a multinomial analysis was performed to determine the association between the severity of asthma and the explanatory variables. The strength of association was determined using odds ratio at 95% confidence interval and P-value < 0.05 was considered to declare significant associations. The results are presented in the form of narrative text, tables, and figures.

### Ethics approval and consent to participate

Permission to conduct this research was granted by the institutional review board, Jimma University, Faculty of Health sciences prior to study initiation, and by the general director of JMC, Ethiopia. Ethical approval was obtained from the institutional review board (IRB) of the Faculty of Health Sciences of Jimma University. During data collection, written informed consent was obtained from head nurse emergency department of the JMC.As this is card review, there is no need to obtain informed consent from patients to participation. All methods were performed in accordance with the relevant guidelines and regulations.

## Results

### Socio-demographic characteristics

Table 1 shows the socio-demographic characteristics of the patients’. Of the 195 patients, 189 fulfilled the eligibility criteria, resulting in a response rate of 96.9%. More than half of the patients were female and over one-third were aged 20–39 years old years. Most patients lived in rural areas and 95 out of 100 patients had at least one comorbidity (Table 1).

**Table 1:** Sociodemographic characteristics of adult patients assisted at the Adult Emergency Department of the Jimma Medical Center, Jimma Town, Ethiopia during 25 July, 2021 to 25 August, 2021.

Variables	Category	Frequency	Percent
Age in years	20–39	73	38.6
	40–59	62	32.8
	≥ 60	54	28.6
Gender	Female	105	55.6
	Male	84	44.4
Residence	Rural areas	103	54.5
	Urban areas	86	45.5
Occupation	Farmer	49	25.9
	Merchant	57	30.2
	Government employees	51	27.0
	Daily laborers	32	16.9
Smoking status	No	133	70.4
	Yes	56	29.6
Comorbidity	No	10	5.3
	Yes (pneumonia, allergic rhinitis, COPD and CVD)	179	94.7

*COPD * chronic obstructive pulmonary diseases, *CVD* cardiovascular diseases.

### Prevalence of symptoms of asthma

Table 2 shows the prevalence of the symptoms of asthma. Most of the patients had wheezing which is the most common symptom experienced by patients admitted to the adult emergency department followed by dyspnea, limited daily activity and coughing (Table 2).

**Table 2:** Clinical presentation of adult patients assisted at the Adult Emergency Department of the Jimma Medical Center, Jimma Town, Ethiopia during 25 July, 2021 to 25 August, 2021.

Asthma symptoms	Category	Frequency	Percent
Wheezing	No	37	19.6
	Yes	152	80.4
Cough	No	77	40.7
	Yes	112	59.3
Shortness of breath	No	50	26.5
	Yes	139	73.5
Limits daily activity	No	69	36.5
	Yes	120	63.5
Rhinitis	No	128	67.7
	Yes	61	32.3
Sinusitis	No	139	73.5
	Yes	50	26.5
Altered mental status	No	148	78.3
	Yes	41	21.7
Cyanosis	No	159	84.1
	Yes	30	15.9

### Severity of asthma

**Figure 1:**
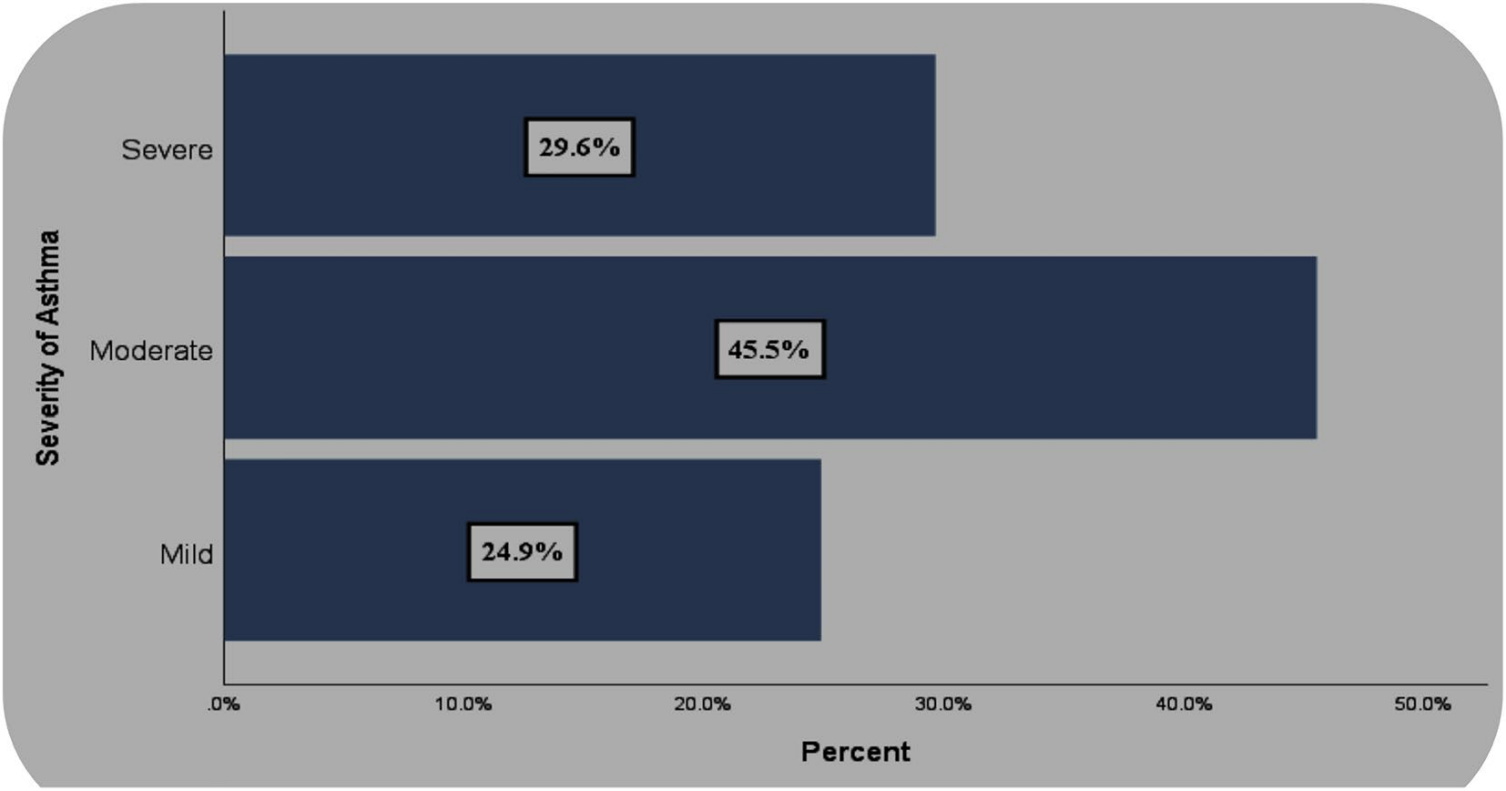
Severity of asthma in adult patients assisted at the Adult Emergency Department of the Jimma Medical Center, Jimma Town, Ethiopia during 25 July, 2021 to 25 August, 2021.

Figure 1 shows the severity of the asthma. Regarding the severity of asthma, more than one-third of patients had moderate asthma followed by severe and mild (Fig. 1).

### Multinomial analysis of factors associated with asthma severity

Table 3 shows multinomial analysis of factors associated with severity of asthma. Gender was associated with moderate asthma as compared to mild asthma. This shows that males have lower odds of moderate asthma than female counterparts. Occupational status was one of influencing factors of severity of asthma indicating merchants and government employees were negatively associated with moderate asthma whereas daily laborers had higher odds of moderate asthma in relation to farmers. Smoking status was positively associated with moderate asthma suggesting that smoking contributed increased odds of moderate asthma.

**Table 3:** Multinomial analysis of factors associated with severity of asthma of adult patients assisted at the Adult Emergency Department of the Jimma Medical Center, Jimma Town, Ethiopia during 25 July, 2021 to 25 August, 2021.

Variables	Category	Severity of asthma		
		Mild^a^	Moderate	Severe
			OR (95%CI)	OR (95%CI)
Age	20–39 years^b^	1	1	1
	40–59 years		0.29 (0.08–1.02)	3.09 (1.68–4.16)*
	≥ 60 years		0.57 (1.56–2.08)	5.7 (2.34–7.07)**
Gender	Female^b^	1	1	1
	Male		0.86 (0.69–0.98)*	1.66 (0.06–1.85)
Residence	Rural^b^	1	1	1
	Urban		0.26 (0.08–1.97)	0.19 (0.04–0.83)**
Occupational status	Farmer^b^	1	1	1
	Merchant		0.3 (0.10–3.06^)^**	0.71 (0.50–1.73)
	Government employee		0.60 (0.55–0.87)**	0.23 (0.02–1.76)
	Daily laborer		3.48 (3.91–141.18)**	5.98 (0.70–8.27)
Smoking status	No^b^	**1**	**1**	**1**
	Yes		4.72 (1.44–15.46)*	1.44 (0.27–6.43)
Comorbidity	No^b^	**1**	**1**	**1**
	Yes		3.54 (0.32–8.86)	4.56 (1.11–8.97)*

*P < 0.05, **P < 0.001.

^a^Reference for dependent variable.

^b^Reference for the category of independent variable.

Significant values are in bold.

Patients’ age was positively associated with severe asthma increased by 3.09 times (95% CI: 1.68–4.16, p < 0.05) in middle ages and 5.7 times (95% CI: 2.34–7.07, p < 0.001) in patients aged ≥ 60 years compared to patients in the age group of 20–39 years. The place of residence affects the severity of asthma. This which means that urban residents have decreased odds of severe asthma in relation to their rural counterparts. It was found that patients with comorbidities had increased odds of severe asthma compared to patients without comorbidities (Table 3).

## Discussion

Asthma is a common, chronic respiratory disease affecting 300 million people worldwide and 50 million people in African^6^. Poor control of asthma is the main cause of emergency-department (ED) access, becoming the strongest determinant of the economic burden of asthma management^19^ continues to increase especially in low-and middle income countries (LMICs), posing a substantial public health threat^1^ including Ethiopia. Early and accurate diagnosis of asthma according to its severity is fundamental for its optimal management^1^. Asthma severity is assessed retrospectively based on the level of treatment required to control symptoms and exacerbations.

In this study, more than one-third of the patients had moderate asthma, suggesting patients were only able to talk in phrases, mentally agitated, and used accessory muscles. The results of the current study indicate that gender, occupational status, and smoking status are associated with moderate asthma. Gender affects the severity of asthma development, with males having decreased odds of moderate asthma. This implies that differences appear to be the product of biological sex differences as well as sociocultural and environmental differences. Biological sex differences include genetic, pulmonary (developmental), and immunologic factors^20^. The differences in asthma severity trends suggest that sex hormones are implicated in asthma pathogenesis, with female sex hormones and their receptors favoring asthma development and male sex hormones and their receptors exerting a protective effect. Some authors have also indicated greater hyper-responsiveness in females than in males and others have highlighted differences between genders in lung capacity^21^.

Furthermore, in low-and middle income countries (LMICs), including Ethiopia different indoor activity patterns might also be associated with differences in exposure between men and women. For example, cooking with gas has been shown to be associated with respiratory symptoms and a small reduction in lung function, and females generally perform most cooking^20^.

Occupational status was a predictor of moderate asthma indicating that daily laborers had increased odds relative to their counterparts. This means that patients who are daily laborers are from low income households, which limits the application of appropriate prevention and control mechanisms for asthma due to low the access to healthcare facility by patients. In addition , these individuals live in low quality housing occupied by such groups, use charcoal as a source of energy and live in rented houses which induce stress resulting in poor medication adherence and symptom exacerbation finally poor asthma control and quality of life^8^.Moreover, daily laborers are financially dependent on their daily job done manually in which they are more likely to be exposed to etiological agents chemicals which in turn results asthma symptoms exacerbation^22^.

Conversely, government employees and merchants were less likely to develop moderate asthma. This may be explained by the fact that government employees and merchants may have a good monthly income they are able to afford for quality care to control asthma as compared to their counterparts. These individuals are educated so that they live in urban areas, which increases the chance of obtaining information from different sources about disease control mechanisms ,such as avoiding contacts with etiologic agents, appropriate use of asthma medications and physical activity to prevent and control comorbidities thus reducing morbidity and mortality from asthma and improving quality of life^23^.

Smoking increased the odds of moderate asthma relative to those who did not smoke. This finding implies that smoking aggravates asthma symptoms and decreases the effects of medication^16^. Moreover, the effect of smoking on the airway such as tobacco smoke flows inward to inflammatory cells, such as neutrophils, lymphocytes, eosinophils, mast cells and macrophages. Various inflammatory mediators are released which including lipids, chemokines, cytokines and growth factors and which cause inflammatory damage and bronchial hyper-responsiveness, a hall mark of asthma^24^.

In this study, participants aged 40–59 and ≥ 60 years and having comorbidities had an increased likelihood odds ratio of while participants residing in urban areas had decreased odds of severe asthma compared to their counterparts. Age seems to influence the severity of asthma.. Asthma is a heterogeneous disease that affects patients from childhood to old age. Aging-related problems such as comorbidities in the elderly. There are also age-related issues leading to decreased disease control such as non-adherence, tobacco use, difficulty in using inhalers and corticosteroid-related side effects that hinder asthma control in different age groups^14^.

Comorbidities affect asthma severity. In the current study, ninety-five percent of the participants had comorbidities. The most common observed comorbidities in this study were pneumonia, allergic Rhinitis, COPD and CVD resulted patients in poorly controlled disease, elevated healthcare costs, reduced work productivity and poor quality of life which puts further strain on already economically burdened families and countries^25^ including Ethiopia.

This means that the presence of comorbidities can complicate the diagnosis and management of asthma which in turn may lead to poor control of asthma. Comorbid conditions can present varying challenges, including diagnostic confusion due to exacerbation of asthma symptoms, therapy for comorbid conditions affecting asthma or therapy for asthma affecting these conditions^26^.This suggests the importance of a detailed categorization of patients with asthma in terms of comorbidities to tailor the best management^27^.

In the current study, urban residents were less likely to have severe asthma than their rural counterparts were. This is in contrast to a study demonstrating that urbanization was associated with asthma attributed to several factors, such as westernized diet, obesity, sedentary lifestyle, outdoor pollutants, and indoor pollutants from increasing industrialization and migration. Alternatively, it has been postulated that this discrepancy is due to the under-diagnoses of allergic conditions due to poor access to medical care among rural residents^13^. Tegegnework, et al. found that urban residents were more likely to develop asthma than were rural residents. This could be explained by the fact that outdoor air in urban areas is highly polluted due to high levels of traffic and industry related emissions which could increase the risk of severe asthma^15^.

Another study found that people residing in rural households that are unclean, use firewood for cooking, crop residual and cow dung cake which leads to indoor air pollution, are more likely to report severe asthma^12^.

This study had several strengths and limitations. To the best of our knowledge, this is the first study in this area to identify the asthma severity. A strong analysis method was used to identify the associated factors. One of the limitations of this study was all patients who were proposed to be included in the study were not involved in the study due to data incompleteness especially for data related to age, comorbidities, address of the patients, a season of the register, and severity level. The main limitation of this study was the inability to assess many risk factors that affect the asthma severity which has greater importance for the prevalence of asthma in different studies. It may not be generalizable beyond the study population because it involves patients at a single center and a small sample size.

In conclusion, this study underlines that forty-six percent of patients had moderate asthma. Being male, merchant and government employees were negatively associated with moderateasthma than their counterparts whereas being daily laborers and smoking were positively associated with moderate asthma. Patients’ age and comorbidities had increased odds of severe asthma compared to their participants their reference category. Urban residents had decreased odds of severe BA in relation to rural counterparts.

Thus JMC should have guidelines for the classification of asthma based on severity to accurately diagnose and manage asthma appropriately. Should provide up-to-date training for ED staff for the proper classification of asthma based on its severity. This also calls for health care providers to give due attention while providing care routine for their patients in accordance to identified factors especially in patients with comorbidities rigorously stratify patients with asthma in terms of comorbidities, to tailor the best management. Further longitudinal studies should be conducted to determine its causes and effects.

## Data Availability

The datasets used in the current study are available from the corresponding author upon reasonable request.

## Abbreviations

COPD: Chronic obstructive pulmonary disease
CVD: Cardiovascular diseases
JMC: Jimma Medical Center
OR: Odds ratio
CI: Confidence interval
ED: Emergency department
LMICs: Low and middle income countries
